# Circulating CD36 Is Reduced in HNF1A-MODY Carriers

**DOI:** 10.1371/journal.pone.0074577

**Published:** 2013-09-12

**Authors:** Siobhan Bacon, Ma P. Kyithar, Jasmin Schmid, Andre Costa Pozza, Aase Handberg, Maria M. Byrne

**Affiliations:** 1 Department of Endocrinology, Mater Misericordiae University Hospital, Dublin, Ireland; 2 Department of Physiology and Medical Physics, Royal College of Surgeons in Ireland, Dublin, Ireland; 3 Department of Clinical Biochemistry, Aalborg University Hospital, Aalborg, Denmark; Sapienza University of Rome, Italy

## Abstract

**Introduction:**

Premature atherosclerosis is a significant cause of morbidity and mortality in type 2 diabetes mellitus. Maturity onset diabetes of the young (MODY) accounts for approximately 2% of all diabetes, with mutations in the transcription factor; hepatocyte nuclear factor 1 alpha (HNF1A) accounting for the majority of MODY cases. There is somewhat limited data available on the prevalence of macrovascular disease in HNF1A-MODY carriers with diabetes. Marked insulin resistance and the associated dyslipidaemia are not clinical features of HNF1A-MODY carriers. The scavenger protein CD36 has been shown to play a substantial role in the pathogenesis of atherosclerosis, largely through its interaction with oxidised LDL. Higher levels of monocyte CD36 and plasma CD36(sCD36) are seen to cluster with insulin resistance and diabetes. The aim of this study was to determine levels of sCD36 in participants with HNF1A-MODY diabetes and to compare them with unaffected normoglycaemic family members and participants with type 2 diabetes mellitus.

**Methods:**

We recruited 37 participants with HNF1A-MODY diabetes and compared levels of sCD36 with BMI-matched participants with type 2 diabetes mellitus and normoglycaemic HNF1A-MODY negative family controls. Levels of sCD36 were correlated with phenotypic and biochemical parameters.

**Results:**

HNF1A-MODY participants were lean, normotensive, with higher HDL and lower triglyceride levels when compared to controls and participants with type 2 diabetes mellitus. sCD36 was also significantly lower in HNF1A-MODY participants when compared to both the normoglycaemic family controls and to lean participants with type 2 diabetes mellitus.

**Conclusion:**

In conclusion, sCD36 is significantly lower in lean participants with HNF1A-MODY diabetes when compared to weight-matched normoglycaemic familial HNF1A-MODY negative controls and to lean participants with type 2 diabetes mellitus. Lower levels of this pro-atherogenic marker may result from the higher HDL component in the lipid profile of HNF1A-MODY participants.

## Introduction

Cardiovascular disease is the major cause of morbidity and mortality in diabetes and is the largest contributor to the direct and indirect cost of managing subjects with type 1 (T1DM) and type 2 diabetes mellitus (T2DM). Heterozygous mutations in the HNF1A gene, encoding the transcription factor hepatocyte nuclear factor-1 alpha, cause autosomal dominant inherited diabetes known as Maturity Onset Diabetes of the Young (MODY). Mutations in the HNF1A gene are the most common cause of MODY in the UK, accounting for approximately 50% of their cases [1]. T2DM is a heterogeneous disease; in contrast HNF1A-MODYdiabetes has a dominant inheritance with high penetrance.

T2DM is characterised by insulin resistance and beta cell failure, whereas HNF1A-MODY patients have been shown to have normal insulin sensitivity [2,3]. The insulin resistance in T2DM is associated with a specific dyslipidaemia; typically of hypertriglyceridaemia, low high density lipoprotein (HDL) and relatively normal low density lipoprotein (LDL). In contrast, previous studies assessing the lipid profiles of HNF1A-MODY patients have demonstrated a favourable profile from a cardiovascular aspect with lower fasting triglyceride levels and comparable HDL levels to normoglycaemic controls [4,5]. There has been no prospective study performed to date on subjects with HNF1A-MODY with regards to macrovascular complications. The data available is therefore limited to retrospective analysis of macrovascular disease in these participants [6].

Macrophage CD36 is believed to play a critical role in the initiation and progression of atherosclerosis through its ability to bind and internalize LDL, thereby facilitating in the formation of foam cells [7,8]. Monocyte/macrophage CD36 is elevated in diabetes, possibly induced by insulin resistance, and it has been proposed that this could partly explain the accelerated atherosclerosis in T2DM.

Elevated *plasma* sCD36 levels have also been reported in obese T2DM, and in pre-diabetic states such as obesity and polycystic ovary syndrome [9,10], and have been proposed to be a marker of insulin resistance and atherosclerosis risk.

To date, sCD36 has been investigated primarily in subjects with insulin resistance. The aim of this study was to determine levels of sCD36 in HNF1A-MODYparticipants. We hypothesised that sCD36 would be lower in this unique population as participants with HNF1A-MODY are relatively insulin sensitive when compared to subjects with T2DM. sCD36 was subsequently correlated with insulin sensitivity, glycaemic control, phenotypic characteristics and biochemical parameters.

## Methods

### Participants

37 participants with HNF1A-MODY diabetes participated in the study. HNF1A mutations included L17H, G207D, P291finsC, S352fsdelG, F426X, P379T, IVS7-6G>A, R200Q/N and E230fsdelGA. In addition, 21 participants with T2DM, who were BMI-matched with the HNF1A-MODY group were recruited from the diabetes outpatients in the Mater Misericordiae University Hospital. These participants were selected because they did not satisfy clinical criteria for testing for MODY, in particular they did not have any significant family history of diabetes and were diagnosed with diabetes at an older age. A further 11 participants, who were family members of the HNF1A-MODY group but negative for the mutation and normoglycaemic formed a control group. The clinical characteristics of all groups analyzed are presented in Table 1. Ethics approval was attained from the ethics committee at the Mater Misericordiae University Hospital. All study participants gave written informed consent to participate in the study.

**Table 1:** Clinical characteristics of subjects.

	***HNF1A-MODY***	***NormoglycaemicHNF1A-MODYnegative***	***T2DM***	***HNF1A-MODYvsHNF1A-MODYnegativecontrols***	***HNF1A-MODYvsT2DM***
n=	37	11	21		
Age (yrs.)	38 (± 16)	30 (±14)	50 (±16)	ns	0.01
Duration (yrs.)	12.3 (±11.5)	na	3.8 (±3.3)	<0.001	0.01
BMI (kg/m^2^)	24.9 (±6.8)	25.4 (± 2.6)	24.2 (±5.3)	ns	ns
SBP (mmHg)	121.6 (±16)	118.9 (± 14.6)	134.1 (±19.4)	ns	0.01
DBP (mmHg)	71.6 (±16)	71 (12.4)	77.8 (11.4)	ns	0.03
Total Cholesterol (mmol/l)	4.3 (± 0.9)	4.7 (± 0.9)	4.1 (± 0.9)	ns	ns
HDL (mmol/l)	1.4 (±0.5)	1.2 (±0.4)	1.1 (± 0.3)	ns	0.03
TG (mmol/l)	1.0 (± 0.7)	1.0 (± 0.5)	1.5 (± 1.0)	ns	0.008
LDL (mmol/l)	2.4 (±0.7)	3 (±0.9)	2.2 (±0.9)	0.03	ns
CD36 (arbitrary units)	0.7 (±0.4)	1.1 (±0.5)	1.0 (±0.5)	0.03	0.05
OGIS (ml/min/m^2^)	359 (±91)	471 (±53.3)	310 (±71)	0.003	ns
HbA1c (%) mmol/l	7.2 (±1.1)	5.2 (±0.2)	7.4 (±1.3)	<0.0001	ns
Fasting insulin (pmol/l)	26.9 (±33)	37.6 (±22)	67.9 (±42)	ns	0.002
hs CRP	0.4 (±0.6)	0.8 (±0.7)	1.9 (±2.2)	ns	<0.0001
ALT(IU/L)	24.6 (±29)	23 (±12)	35.4 (±18)	ns	ns

### Genetic Analysis

Analysis of the HNF1A gene was performed by polymerase chain reaction (PCR) amplification of highly purified genomic DNA, followed by semi-automated unidirectional DNA sequencing of all exons including the highly conserved flanking intronic sequences of the exon-intron splice junctions. Genetic analysis was performed by integraGen GmbH (Bonn, Germany) in 2000-2007 and the Molecular Genetic Laboratory (Exeter) from 2008-2012.

### Clinical and laboratory measurements

All participants were BMI matched. Exclusion criteria included pregnancy. All participants underwent a full clinical assessment, including a full medical history and physical examination. Anthropometric measurements including weight, height, and body mass index (BMI) were obtained. A detailed neurological history was performed and a physical examination focusing on the signs and symptoms of distal symmetrical sensorimotor polyneuropathy. An eye fundus examination was performed using retinal photography. Urine samples were analysed for urinary glucose and urinary microalbumin/creatinine ratio (ACR). Microalbuminuria was considered to be present if the ACR was greater than 3.4g/mol. A history of macrovascular disease i.e. coronary heart disease (myocardial infarction/angina/coronary bypass grafting or percutaneous coronary intervention), cerebrovascular disease (ischaemic stroke) or peripheral vascular disease was recorded from medical history and hospital records.

A 75 g OGTT was performed on participants after a 12-h overnight fast with measurement of glucose, insulin and C-peptide at baseline and at 30 minute intervals for 120 minutes to determine the degree of glucose tolerance and insulin secretory response. In patients with diabetes, oral hypoglycaemic agents were stopped at least 48-h before the OGTT while, in those taking insulin, long-acting insulin therapy was stopped for 24-h and short-acting insulin stopped for 12-h prior to OGTT. The diagnostic criteria for the American Diabetes Association were used to define the degree of glucose tolerance. The oral glucose insulin sensitivity (OGIS) was calculated as previously described [11].

Blood samples were drawn for the measurement of HbA_1c_, fasting lipids, full blood count, thyroid function, renal and liver profiles, glutamic acid decarboxylase (GAD65) auto antibodies, and pancreatic islet cell auto antibodies (ICA). In addition a blood sample for sCD36 and hsCRP was drawn.

### Assays

All laboratory analyses were performed with commercially available standardized methods. The plasma glucose concentration was measured using Beckman Synchron DXC800 (Beckman Instruments Inc, Brea, USA). HbA_1c_ was determined using high performance liquid chromatography (Menarini HA81-10, Rome, Italy). Insulin and C-peptide were analyzed using Immulite 2000 immunoassay (Siemens Healthcare Diagnostics, Deerfield, IL, USA). GAD antibodies were analysed using competitive fluid-phase radioimmunoassay by the neurosciences group in John Radcliffe Hospital in Oxford, and ICA by indirect immunofluorescence test by the Supra-Regional Protein Reference Unit and Department of Immunology in Sheffield, UK.

### Plasma sCD36

sCD36 was measured using an in-house enzyme-linked immunoassay (ELISA). A pool of EDTA plasma was applied in seven dilutions and used to produce a standard concentration curve. Internal controls were run in quadruplicate on each plate. Runs were accepted if the controls were within ±2 SD from mean, and most were within 1 SD. The intra-assay coefficient of variation (CV) was 6%. Log-transformed standard curves were linear. A few measurements were outside the standard curve range and were calculated by extrapolation. sCD36 was measured in fasting samples.

### Measurement of serum hsCRP levels

Serum hsCRP levels were measured using particle enhanced immunonephelometry assay (CardioPhase® hsCRP, Siemens) on a Siemens BN II analyzer (Siemens Healthcare Diagnostics, Deerfield, IL, USA). A typical limit for detection of hsCRP was 0.175mg/L for measurements performed using a sample dilution of 1:20. A coefficient of variation at the concentration 0.41mg/L was 7.6%. We considered that hsCRP values >10mg/l were likely to represent an inflammatory response in line with previous studies [12-14]. We therefore performed two separate analysis approaches, one in which we included (termed ‘all patients’), and one in which we excluded (termed ‘without extreme CRP’) the 2 HNF1A-MODY patients with serum hsCRP values of >10 mg/l.

### Statistical analysis

Clinical and biochemical data are expressed as mean ± SD. Statistical analysis was performed using SPSS statistical software package for Windows, version 20.0 (SPSS, Chicago, IL, USA). The significance of the difference between 2 groups was determined by Mann-Whitney U test (non-parametric clinical data) or t-test. For comparisons of more than 2 groups, Kruskal-Wallis test was applied. Differences were considered significant at p <0.05. Fisher exact test was applied for aspirin and statin use.

## Results

The clinical characteristics of the lean HNF1A-MODY, the BMI-matched T2DM and normoglycaemic HNF1A-mutation negative family member groups are shown in Table 1.

Of the 37 HNF1A-MODY carriers with diabetes, 9 subjects (24.3%) had diabetic retinopathy, with proliferative retinopathy in one subject (2.7%). Only one of the subjects had evidence of microalbuminuria.

There was no personal history of myocardial infarction or ischaemic stroke in the HNF1A-MODY subjects, 3 had significant peripheral vascular disease, 2 requiring femoral popliteal bypass grafting.

**Figure 1:**
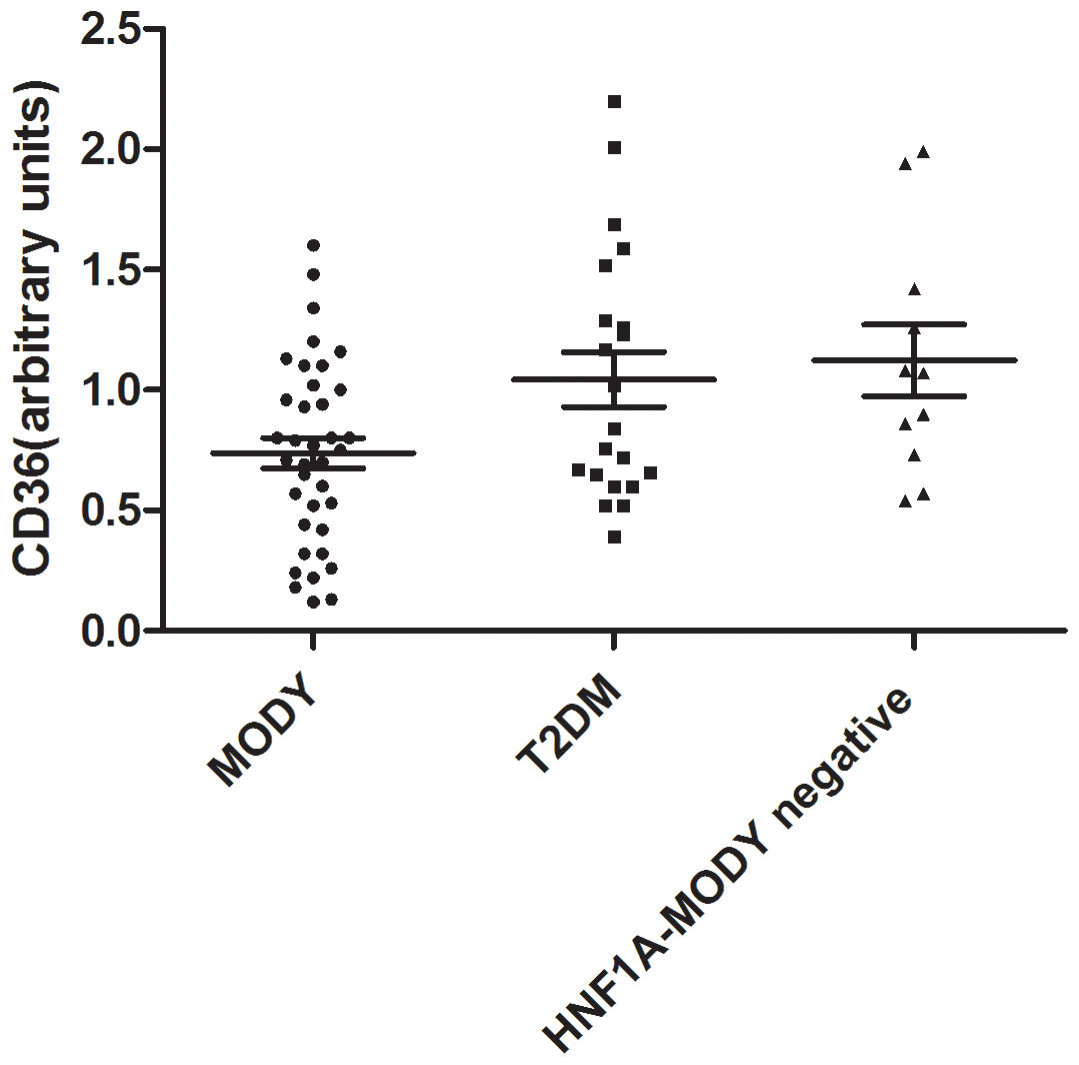
sCD36 (arbitrary units) in HNF1A-MODY (black circle), T2DM (black square) and HNF1A-MODY negative normoglycaemic control (black triangle). Table footnote; BMI=Body Mass Index, SBP=Systolic Blood Pressure, DBP=Diastolic Blood Pressure, HDL=High density Lipoprotein, TG=Triglycerides, LDL=Low Density Lipoprotein, OGIS=Oral Glucose Insulin Sensitivity Index. hsCRP=high sensitivity C-reactive protein.ALT=Alanine aminotransferase. ns=not significant.na=not applicable

Levels of sCD36 were significantly lower in participants with HNF1A-MODY when compared to normoglycaemic HNF1A-MODY negative family controls (0.7 (±0.4) vs 1.1 (±0.5), p=0.03). Similarly, levels of sCD36 were significantly lower when compared to participants with T2DM (0.7 (±0.4) vs 1.0 (±0.5), p=0.05). This is further illustrated in Figure 1. When multivariate analysis was performed accounting for age and duration of diabetes status the levels of sCD36 remain significantly different between HNF1A-MODY participants and normoglycaemic HNF1A-MODY negative controls. Insulin sensitivity as determined using OGIS was higher in participants with HNF1A-MODY when compared to the BMI-matched participants with T2DM, however, this did not reach statistical significance (359 ±91 vs 310±71 ml/min/m^2^)

Levels of sCD36 in HNF1A-MODY did not correlate with age, duration of diabetes or glycaemic control as determined using HbA_1c_. Similarly there was no correlation noted with insulin sensitivity as determined using OGIS or hsCRP. There was a positive correlation noted with ALT (rho=0.37, p=0.04).

There was significantly less statin (21.6% vs. 61.9%, p=0.001) and aspirin therapy (30% vs. 60.8%, p=0.01) prescribed in the HNF1A-MODY group compared to the T2DM group.

## Discussion

### HNF1A-MODY participants lack features of the metabolic syndrome

HNF1A-MODY patients have been shown in this current study to have a better metabolic profile compared to BMI-matched T2DM controls. They have significantly lower triglyceride levels, higher HDL levels with less hypertension. This is similar to what has been reported previously in the UK [5].

### CD36 is significantly reduced in HNF1A-MODY participants

In keeping with the absence of the metabolic syndrome features, soluble CD36 levels are significantly lower in the HNF1A-MODY participants when compared to the normoglycaemic HNF1A-MODY negative family control group and the T2DM group. CD36, a 88kDa, highly glycosylated transmembrane protein is well established to facilitate the uptake of long chain fatty acids (LCFA) into adipocytes, the heart and skeletal muscle. CD36 is known from its function in macrophages as a scavenger receptor for oxidised LDL. sCD36 clusters with markers of insulin resistance and is progressively related to the severity of insulin resistance and atherosclerosis in the human population [10,15-17]. A previous study demonstrated that in a healthy group a positive relationship was also noted between elevated sCD36 and carotid intima medial thickness. The findings proposed that elevated sCD36 has the potential to be an important marker of atherosclerosis [18]. In other, small-scale studies these relationships were only present in insulin resistant individuals [9,10]. In the current study, sCD36 is low in the HNF1A-MODY population when compared to family controls and T2DM participants. We can speculate that HNF1A may play a role in the regulation of sCD36; however, further study with a larger population size is required to confirm its potential use as a marker of atherosclerosis.

Our current study, in keeping with previous findings, has demonstrated that individuals with HNF1A-MODY have significantly lower levels of hsCRP when compared to individuals with T2DM [19]. There is a plausible biological reason for this as the promoter of the CRP gene has 2 binding sites for hepatocyte nuclear factor 1A [19]. Elevated levels of high sensitivity C-reactive protein (hsCRP) has been shown to be associated with increased cardiovascular risk

We did not find a significant correlation between sCD36 and insulin sensitivity in the participants with HNF1A-MODY when determined using OGIS. OGIS has been shown to be analogous to the assessment of insulin sensitivity using the hyperinsulinaemic euglycaemic clamp technique and is more practical for use in a clinical setting [11]. This is the first study to look at sCD36 in HNF1A-MODY. As HNF1A-MODY is rare in comparison to T2DM, the sample size studied maybe affecting correlation significance.

We have demonstrated a positive correlation between sCD36 and ALT. ALT levels did not differ significantly between the 3 groups studied. Elevated aminotransferases are surrogate markers for liver fat content. In apparently healthy cohorts an elevated ALT is associated with hepatic insulin resistance [20]. Subjects with HNF1A-MODY, as aforementioned are relatively insulin sensitive when compared to subjects with T2DM; however, it is known that altered liver function can occur in forms of MODY [21]. HNF1A-MODY is highly expressed in the liver and is involved in the regulation of many liver specific genes [21].

In the HNF1A- MODY group studied there was no personal history of myocardial infarction or ischaemic stroke. The 3 subjects who had peripheral vascular disease were all smokers.

In this current study, the T2DM population had lower sCD36 levels than previously reported, however this group was selected to be BMI-matched with the lean HNF1A-MODY population and therefore not comparable to previously studied patients. In addition, the majority of the T2DM group was on statin and aspirin therapy; both of which are known to reduce CD36 levels [22] therefore it is likely that the levels noted in this study for the T2DM group are lower than expected.

### CD36 is a cellular receptor for HDL

HDL plays an important role in cholesterol homeostasis removing cholesterol from peripheral tissues such as vessel walls. A mouse model with a targeted mutation in the CD36 gene has a significant increase in HDL cholesterol compared to wild type littermates [23,24]. A recent study proposed a deficiency of CD36 promoting HDL formation. A lack of CD36 may be associated with an increase in hepatic cholesterol and phospholipid efflux and stimulated hepatic secretion of apolipoproteins. These changes may represent a mechanism that mediates an increase in plasma HDL cholesterol [23]. A further study recently concluded that CD36 mediates uptake of HDL by tissues particularly by the liver and the adrenal glands [25] and that through these two mechanisms; increased HDL biosynthesis and reduced HDL catabolism an increase in the steady state plasma HDL cholesterol is achieved in the CD36 deficient mice.

In conclusion, HNF1A-MODY carriers have significantly lower levels of sCD36, a marker which is known to cluster with insulin resistance and atherosclerotic plaque development. In accordance with previous studies we have demonstrated that HNF1A-MODY carriers have lower levels of hsCRP when compared to subjects with T2DM. This is the first study to measure sCD36 in the HNF1A-MODY population. We have also provided a possible relationship between low sCD36 levels and the high HDL levels noted in these patients which perhaps is conferring a protective mechanism in HNF1A-MODY carriers against macrovascular disease. The marker sCD36 warrants further investigation in a larger population of HNF1A-MODY carriers and control subjects.
